# Faecal calprotectin for the diagnosis of bowel inflammation in patients with rheumatologic diseases: what is new in the last 5 years (2020-2025)?

**DOI:** 10.1016/j.ero.2026.100206

**Published:** 2026-06-25

**Authors:** Caroline Morizot, Bénédicte Caron, Jean-Yves Jouzeau, David Moulin, Patrick Netter, Sailish Honap, Damien Loeuille, Laurent Peyrin-Biroulet

**Affiliations:** 1 Department of Rheumatology, Nancy University Hospital, Nancy, France; 2 Centre National de la Recherche Scientifique, Ingénierie Moléculaire Cellulaire et Physiopathologie (IMoPA), Université de Lorraine, Vandœuvre-lès-Nancy, France; 3 Department of Gastroenterology, Nancy University Hospital, Nancy, France; 4 Institut National de la Santé Et de la Recherche Médical, Nutrition-Génétique et Exposition aux Risques Environnementaux (NGERE), Université de Lorraine, Vandœuvre-lès-Nancy, France; 5 Department of Clinical Pharmocology and Toxicology, Nancy University Hospital, Nancy, France; 6 Department of Gastroenterology, St George’s University Hospital National Health Service Foundation Trust, London, UK; 7 School of Immunology and Microbial Sciences, King’s College London, London, UK

## Abstract

**Objectives:**

This study updated the evidence (2020-2025) on the diagnostic and prognostic value of faecal calprotectin (FC) in rheumatologic diseases, focusing on spondyloarthritis (SpA), rheumatoid arthritis, and fibromyalgia syndrome.

**Methods:**

A systematic search of PubMed, Web of Science, and Cochrane databases (January 2020-August 2025) was performed. Eligible studies included adults with established rheumatologic diseases reporting FC levels and their associations with gastrointestinal symptoms, endoscopic or histologic inflammation, disease activity, or incident inflammatory bowel disease.

**Results:**

In SpA, a substantial proportion of patients exhibited elevated FC levels, ranging from ∼25% to 70% depending on study design and thresholds (typically 50-250 µg/g). Several studies demonstrated concordance between elevated FC and microscopic or macroscopic intestinal inflammation. FC was frequently associated with gastrointestinal symptoms and, in some cohorts, correlated with disease activity indices and inflammatory burden. Medication exposure emerged as a key confounder, with nonsteroidal anti-inflammatory drugs associated with higher FC levels and biologic therapies generally associated with lower values. In rheumatoid arthritis, evidence remains limited but suggests that FC may be elevated in early or active disease and decrease with treatment. In fibromyalgia, limited data indicate elevated FC in symptomatic subgroups, although specificity for inflammatory processes is uncertain.

**Conclusions:**

FC is a promising noninvasive biomarker for detecting subclinical intestinal inflammation in rheumatologic diseases, particularly SpA. However, heterogeneity in study design, thresholds, and confounding factors limits its clinical applicability.

**KEY MESSAGES**

**WHAT IS ALREADY KNOWN ON THIS TOPIC**

• Subclinical intestinal inflammation is a common feature of spondyloarthritis (SpA) and may contribute to disease pathogenesis.
• Fecal calprotectin (FC) is a validated biomarker of intestinal inflammation and is routinely used in inflammatory bowel disease (IBD).
• Previous studies have suggested that elevated FC levels are associated with gut inflammation and disease activity in patients with SpA.
• The clinical significance of FC in other rheumatic conditions, such as rheumatoid arthritis (R1) and firbomyalgia syndrome (FMS), remains less clearly established.

**WHAT THIS STUDY ADDS**

• This updated systematic reviex summarizes the evidence on FC across rheumatic diseases, includins SpA, RA, and FMS, with a focus on studies published between 2020 and 2025.
• Recent evidence further supports the association between FC levels anf objective markers of intestinal inflammation and gut involvement in SpA.
• Data in RA and FMS remain limited and heterogeneous, highlighting important gaps in current knowledge.
• Significant variability in study design, FC assayx, and diagnostic thresholds limits comparisons between studies and diseases.

**HOW THIS STUDY MIGHT AFFECT RESEARCH, PRACTICE, OR POLICY**

• FC may help clinicals identify SpA patients who could benefit from further gastrointestinal evaluation.- Harmonization of FC measurement methods and clinically relevant disease-specific thresholds is required before broader clinical implementation.
• Future prospective studies are needed to better define the diagnostic, prognostic, and therapeutic utility of FC across different rheumatic diseases, as well as its role in predictind progression to overt IBD.

Alt-text: Unlabelled box dummy alt text

## INTRODUCTION

Rheumatologic diseases such as spondyloarthritis (SpA), psoriatic arthritis (PsA), rheumatoid arthritis (RA), and, more recently, fibromyalgia syndrome (FMS), have increasingly been linked to gastrointestinal (GI) manifestations, many of which remain subclinical. The concept of the gut–joint axis has become a central framework to explain how intestinal dysbiosis and mucosal immune activation contribute to systemic inflammation and musculoskeletal disease. This interplay is most evident in SpA, where microscopic bowel inflammation is documented in up to 5% to 60% of patients, and overt inflammatory bowel disease (IBD) develops in ∼5% to 10% during the disease course [1,2].

Faecal calprotectin (FC), a calcium-binding protein released predominantly by activated neutrophils, is a noninvasive biomarker widely validated in IBD for detecting intestinal inflammation and predicting relapse or therapeutic response [3]. Its utility in rheumatology, however, is still being defined. Early evidence suggests that FC may detect occult bowel inflammation in patients with SpA and related conditions, even in the absence of GI symptoms [[4], [5], [6]].

The 2020 systematic review by Fauny et al [4] synthesised the available literature on FC in SpA and ankylosing spondylitis (AS) [4]. Across 7 studies (875 patients), elevated FC levels were observed in 21% to 71% of cases, strongly correlating with both macroscopic and microscopic intestinal inflammation. Thresholds of 132 and 266 µg/g were reported as predictive for future IBD development. Some studies also noted associations between FC and systemic inflammatory markers or disease activity indices (Ankylosing Spondylitis Disease Activity Score [ASDAS], Bath Ankylosing Spondylitis Disease Activity Index [BASDAI], and Bath Ankylosing Spondylitis Functional Index [BASFI]), although these findings were inconsistent. Importantly, confounding factors such as nonsteroidal anti-inflammatory drug (NSAID) use or exposure to biologic therapies complicated interpretation.

Since then, numerous studies published between 2020 and 2025 have expanded this field, investigating the role of FC across different rheumatologic conditions. These include its ability to reflect subclinical gut inflammation, its association with disease activity, and its potential to predict the onset of IBD in SpA. Moreover, FC has been evaluated in RA and even in FMS, suggesting that intestinal inflammation may contribute to systemic symptoms across a wider spectrum of rheumatologic diseases than previously recognised.

The objective of this review is therefore to synthesise evidence published between January 2020 and August 2025 on the role of FC as a biomarker in rheumatologic diseases. Specifically, we aimed the following:

1. to evaluate the diagnostic accuracy of FC for identifying subclinical intestinal inflammation in SpA and related diseases;
2. to assess the prognostic value of FC in predicting IBD development;
3. to explore associations between FC and clinical disease activity or extra-articular manifestations; and
4. to identify limitations, confounding factors, and priorities for future research on the integration of FC into routine rheumatology practice.

## METHODS

### Literature search strategy

A comprehensive literature search was conducted across PubMed, Web of Science, and the Cochrane Library to identify relevant studies published between January 2020 and August 2025. The search strategy combined keywords and Medical Subject Headings (MeSH) terms related to calprotectin and rheumatologic diseases, as follows:

• ‘faecal calprotectin’ OR ‘faecal calprotectin’ OR ‘calprotectin’
• AND
• ‘spondyloarthritis’ OR ‘axial spondyloarthritis’ OR ‘ankylosing spondylitis’ OR ‘psoriatic arthritis’ OR ‘rheumatoid arthritis’ OR ‘fibromyalgia’.

Additional articles were identified by screening reference lists of included studies and relevant reviews. Only full-text articles were considered.

### Inclusion and exclusion criteria

Eligible studies met the following criteria:

1. Population: adult patients (≥18 years) with a confirmed diagnosis of a rheumatologic disease (SpA, axial spondyloarthritis [axSpA], PsA, AS, RA, and FMS).
2. Intervention: measurement of FC.
3. Outcomes: associations of FC with GI symptoms, subclinical intestinal inflammation (endoscopic or histologic), disease activity indices, or development of IBD.
4. Study design: prospective or retrospective observational studies, clinical trials, or systematic reviews with original data.

Exclusion criteria were as follows: paediatric populations; case reports, editorials, or narrative reviews without original data; studies lacking explicit FC measurement; research published only as conference abstracts without sufficient data for extraction (unless providing novel and relevant information, eg, early-RA studies).

### Study selection

A reviewer (CM) screened all titles and abstracts for eligibility. Full texts of potentially relevant articles were then assessed. Discrepancies were resolved by consensus or, when necessary, by consultation with a second reviewer.

### Data extraction

For each included study, the following data were systematically extracted:

• first author, year of publication, country;
• study design and sample size;
• patient population (disease subtype, age, and sex distribution);
• method of FC measurement and thresholds applied;
• correlation with endoscopic and/or histologic findings;
• association with disease activity indices (eg, ASDAS, BASDAI, DAS-28, Disease Activity Score 28 [DAS-28]);
• relation to GI symptoms or development of IBD; and
• key results and conclusions.

Data were tabulated following the structure described by Fauny et al [4] to allow direct comparison. Separate tables were constructed for SpA, RA, and FMS cohorts. Because the Assessment of SpondyloArthritis international Society (ASAS) criteria encompass both axial and peripheral SpA and several included cohorts applied classification criteria for psoriatic arthritis criteria, patients with PsA were captured within the broader SpA category when reported in the original studies.

### Data synthesis

Given the heterogeneity of study designs, populations, and FC thresholds, a formal meta-analysis was not systematically performed. Instead, a narrative synthesis was conducted, supported by structured summary tables. When available, quantitative findings (sensitivity, specificity, and predictive values) were reported.

A formal pooled risk-of-bias or diagnostic accuracy assessment was not performed, as diagnostic performance was explored descriptively across heterogeneous observational studies rather than quantitatively compared. Diagnostic accuracy was evaluated descriptively based on reported associations between FC levels and endoscopic or histologic findings, rather than through pooled estimates or formal comparative accuracy analyses.

## RESULTS

The present review specifically focuses on studies published between 2020 and 2025, which were not included in the systematic review by Fauny et al [4] and, therefore, provides an updated synthesis of more recent evidence. From an initial pool of 362 studies, 17 met the inclusion criteria for this review (Figure). The studies varied in design but collectively provided valuable insights into the utility of FC across different rheumatologic conditions.

**Figure:**
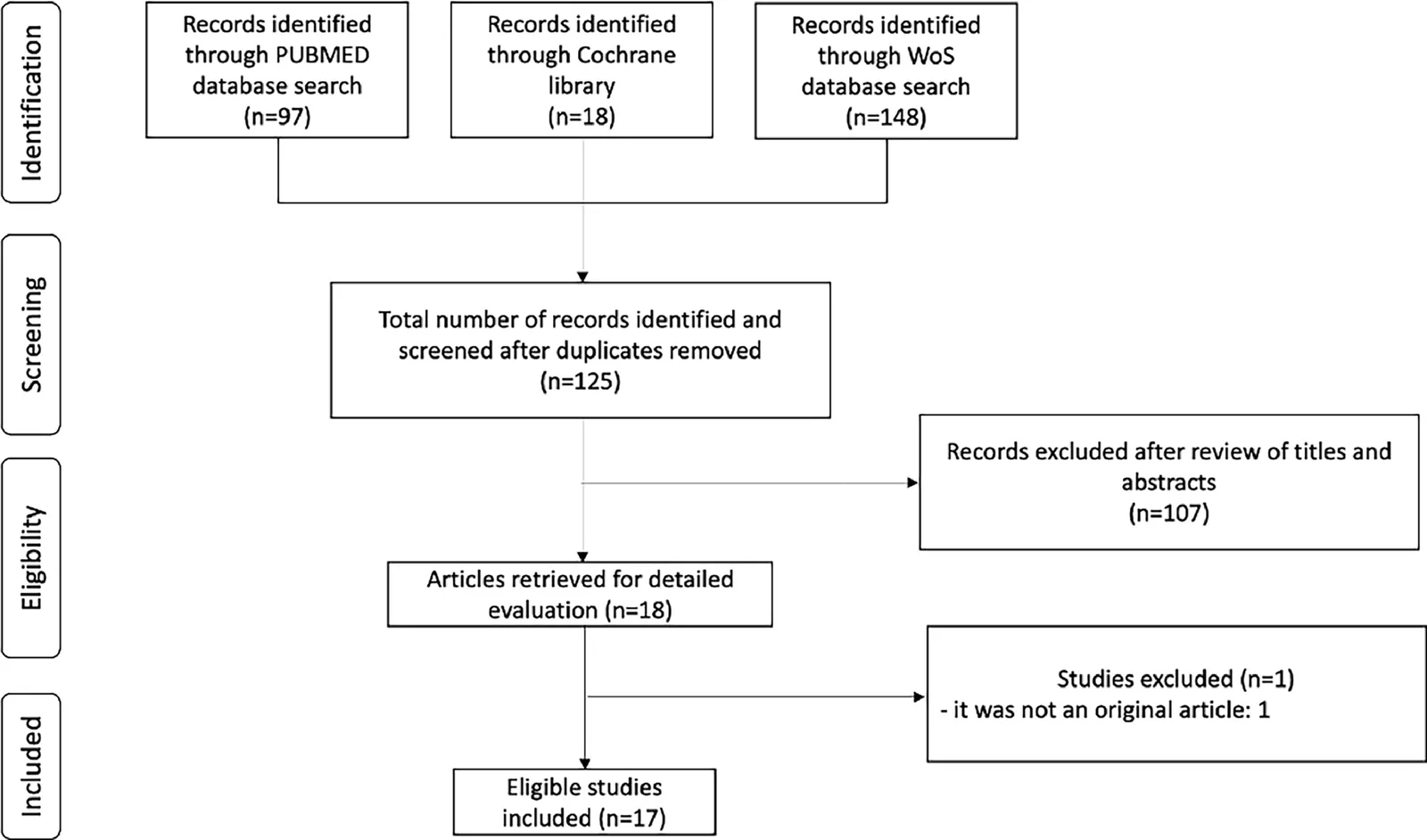
Preferred Reporting Items for Systematic reviews and Meta-Analyses (PRISMA)-style flowchart of study selection process (2020-2025). WoS, Web of Science.

### SpA and axSpA

Across studies, FC was measured either as part of systematic screening strategies in SpA cohorts or in patients presenting with GI symptoms, reflecting real-life heterogeneity in clinical practice (Table 1, Table 2) [[7], [8], [9], [10], [11], [12], [13], [14], [15], [16], [17], [18], [19], [20]]. Across 4 key SpA studies reporting prevalence estimates, a substantial proportion of patients displayed elevated FC, although prevalence varied depending on thresholds. Gutiérrez-Sánchez et al [7] found high FC (>43.2 µg/g) in 24.4% of patients, with diarrhoea and abdominal pain strongly associated with elevated levels. Similarly, De Avila et al [13] reported 27% of patients with SpA and elevated FC, including 16.2% with high values >90 µg/g. In the study by Badran et al [10], 71% of patients had abnormal levels, with endoscopy and histology confirming intestinal inflammation in up to 40% of cases. Campos et al [12] established a cutoff of 96 µg/g as predictive of microscopic bowel inflammation, with 73% sensitivity and 69% specificity.

**Table 1:** Study characteristics—SpA

Reference, year	Country	Design	Aim of the study	Rheumatologic disease definition	Controls	No. of patients	Age (y)^a^	Male (%)	FC assay	Timing vs activity	GI Symptoms	Endoscopy
Gutiérrez-Sánchez et al [7], 2023	Colombia	Interventional	Apply IBD screening criteria in SpA without IBD and correlate with endoscopy/activity	ASAS and ESSG	No	82	NA	55	Quantum Blue	Screening	Yes	Yes
Gazim et al [8], 2023	Brazil	Cross-sectional	Compare FC in AAU with/without SpA	ASAS	No	28	34-51 (by group)	60	ELISA	At flare visit	Not reported	No
Arzuaga-Hernández et al [9], 2025	Colombia	Cross-sectional	Assess GI and psychological symptoms with activity and function in SpA	ASAS	No	98	42 ± 10	58	ELISA	At inclusion	Yes	Yes
Badran et al [10], 2024	Egypt	Cross-sectional	Screen for silent IBD in SpA	ASAS (axSpA and PsA)	No	41	40 (23-58)	60	ELISA	Cross-sectional	Yes	Yes
Brough et al [11], 2024	UK	Cross-sectional	Evaluate stool calprotectin and Th17 cytokines in axSpA	ASAS	Yes (91 HC)	53	NA	NA	ELISA	At inclusion	No	No
Campos et al [12], 2022	Brazil	Cross-sectional	Assess utility of FC for endoscopic/histologic bowel inflammation in SpA	ASAS and CASPAR	No	65	49 ± 12	54	Quantum Blue	Cross-sectional	Not stated	Yes
De Avila et al [13], 2024	Colombia	Cross-sectional	Correlate FC with DCE colonoscopy and histology in SpA without IBD	ASAS and ESSG	No	180	45 ± 10	57	ELISA	Cross-sectional	Not stated	Yes
Emad et al [14], 2022	Spain	Cross-sectional	Diagnostic performance of FC; determine optimal cutoff	ASAS and ESSG	Yes (50 HC)	135	∼42	NA	ELISA	Cross-sectional	Not stated	Not stated
Ercalik et al [15], 2021	Turkey	Cross-sectional	Prevalence of intestinal inflammation; correlation with treatment and disease	ASAS (AS) and ACR (RA)	Yes (49 NIRD)	194	44 ± 13	52	ELISA	Cross-sectional	Not stated	Not stated
Kang et al [16], 2021	Korea	Cross-sectional	Compare FC across manifestations and factors associated with increases in axSpA	ASAS (imaging arm)	No	190	NA	NA	ELISA	Cross-sectional	Not stated	Not stated
Ma et al [17], 2020	China	Meta-analysis	Evaluate FC and serum calprotectin in SpA	Various	Yes (275 HC)	197	NA	NA	Mixed	Aggregated	NA	NA
Sarsilmaz et al [18], 2024	Turkey	Cross-sectional	Assess FC usability as marker of disease activity in SpA	ASAS and CASPAR	No	100	44 ± 11	62	CalFast XT	Cross-sectional	Not stated	Not stated
Sayed et al [19], 2020	Egypt	Cross-sectional	Investigate FC and LCN-2 in subclinical gut inflammation in axSpA	ASAS	Yes (50 HC)	15	38 ± 8	47	ELISA	Cross-sectional	Not stated	Not stated
Shedid et al [20], 2020	Egypt	Cross-sectional	Compare serum and faecal calprotectin in axSpA and their relation to activity/imaging	ASAS (40 axSpA)	Yes (18 HC)	58	40 ± 12	58	ELISA (CALPRO)	Cross-sectional	Not stated	Not stated

a Age is given as mean ± SD or median (range). AAU, Acure Anterior Uveitis; ACR, American College of Rheumatology; ASAS, Assessment of SpondyloArthritis international Society; ASDAS, Ankylosing Spondylitis Disease Activity Score; axSpA, axial spondyloarthritis; AS, ankylosing spondylitis; CASPAR, classification criteria for psoriatic arthritis; DCE, Dye-based ChromoEndoscopy; ELISA, enzyme-linked immunosorbent assay; ESSG, European Spondyloarthropathy Study Group; FC, faecal calprotectin; GI, gastrointestinal; HC, healthy control; IBD, inflammatory bowel disease; NA, not applicable; PsA, psoriatic arthritis; RA, rheumatoid arthritis; SpA, spondyloarthritis.

**Table 2:** Summary of studies on faecal calprotectin in SpA (2020-2025)

Reference, year	Normal FC values (µg/g)	FC values in SpA	FC threshold (µg/g)	Microinflammation	Macroinflammation	GI aymptom association	Endoscopic/histologic correlation	Disease activity correlation	NSAIDs/biologics effect	Key findings
Gutiérrez-Sánchez et al [7], 2023	<43.2	24.4%, >120 ng/mL; 14.6%, >250 ng/mL	>100	NA	NA	Yes (diarrhoea, pain)	NA	NA	NA	Elevated FC (24.4%); diarrhoea and pain linked to higher FC and early IBD signs
Gazim et al [8], 2023	<50	Median, 101 vs 9 µg/g controls	>50	NA	NA	NA	Not reported	NA	NA	Higher FC in SpA+uveitis vs controls; no clear link with uveitis itself
Arzuaga-Hernández et al [9], 2025	<43.2	Median, 54.8 (IQR, 43-130); 29.7%, >120	>43.2	Yes (72.7% histologic)	Yes (51.5% macroscopic)	Yes	Strong correlation (macroscopic + histology)	Yes (BASDAI, BASFI, ASDAS)	NS	FC associated with GI symptoms, depression, and disease activity
Badran et al [10], 2024	<50	71%, more than cutoff; mean, 229-452 µg/g (axSpA vs PsA)	∼76	41.5% histology abnormal	14.6% colonoscopy abnormal	Yes	Yes (macroscopic + histology)	No significant correlation	Biologics ↓ FC	Elevated FC in 71% SpA; histologic lesions common even with low cutoffs
Brough et al [11], 2024	NA	Higher vs healthy	NA	NA	NA	No	No	NS	NS	FC + Th17 cytokines elevated in axSpA stool vs healthy controls
Campos et al [12], 2022	<50	Mean, ∼250 µg/g (±291)	∼96	58.4%	20%	NA	Strong correlation (*P* < .001)	No correlation (BASDAI/ASDAS)	NS	FC >96 µg/g predicts histologic inflammation (73% sensitivity; 67% specificity)
De Avila et al [13], 2024	<43.2	Mean, 64.6 (44-138); 27%, elevated; 16.2%, >250	>90	70.3% mucosal alterations	21.6% rectal/45.9% ileum	Yes (abdominal pain)	Yes (chromocolonoscopy + histology)	Yes (BASDAI, BASFI, ASDAS-CRP)	NS	High FC associated with ileal erosions and disease activity
Emad et al [14], 2022	NA	Higher mean FC in SpA vs USpA and controls	NA	NA	NA	NA	NA	NA	NA	Higher mean FC in SpA vs USpA and controls
Ercalik et al [15], 2021	<50	Mean—AS, 24.9; RA, 17.3; no difference vs controls	>50	NA	NA	Patients with GI symptoms excluded	No	No correlation (BASDAI/DAS–28)	↑ FC in NSAID users; no biologics effect	FC higher in AS vs RA; not linked to disease activity
Kang et al [16], 2021	NA	18% with elevated FC	NA	NA	NA	Yes	No	Yes (ASDAS, ESR, CRP, joints)	NA	Elevated FC correlated with systemic inflammation markers
Ma et al [17], 2020	NA	Pooled ↑ FC in SpA vs controls (OR, 6.22)	NA	NA	NA	Yes	Pooled correlation with histology	Yes (BASDAI, BASFI, CRP, ESR)	NA	FC significantly higher in SpA vs controls
Sarsilmaz et al [18], 2024	<70	Median, 72.5 µg/g (range, 49-1006 µg/g)	>70	NA	NA	GI symptoms excluded	No	Yes (ESR, ASDAS-ESR only)	↓ FC positivity in NSAID users	Modest link between FC and ESR/ASDAS-ESR
Sayed et al [19], 2020	NA	Mean: SpA, 142 vs 25 µg/g controls	NA	Yes (colonic + ileal)	NA	NA	Yes (biopsies)	Yes (ASDAS-ESR, CRP, BASDI)	NA	FC significantly higher in SpA vs controls; correlated with activity scores
Shedid et al [20], 2020	>50	Median, ∼402 vs 134 µg/g controls	>50	NA	NA	No difference	MRI correlation (sacroiliitis)	Yes (ASDAS, function scores)	No difference	FC associated with higher disease activity and MRI sacroiliitis

ACR, American College of Rheumatology; ASDAS, Ankylosing Spondylitis Disease Activity Score; axSpA, axial spondyloarthritis; AS, ankylosing spondylitis; BASDAI, Bath Ankylosing Spondylitis Disease Activity Index; BASFI, Bath Ankylosing Spondylitis Functional Index; CRP, C-reactive protein; DAS-28, Disease Activity Score 28; ESR, erythrocyte sedimentation rate; FC, faecal calprotectin; GI, gastrointestinal; IBD, inflammatory bowel disease; MRI, magnetic resonance imaging; NA, not applicable; NSAID, nonsteroidal anti-inflammatory drug; PsA, psoritic arthritis; RA, rheumatoid arthritis; SpA, spondyloarthritis; USpA, undifferentiated spondyloarthritis.

Importantly, endoscopic and histologic correlations were consistently demonstrated: De Avila et al [13] reported ileal mucosal lesions in nearly half of patients with SpA and high FC, whereas Arzuaga-Hernández et al [9] observed mucosal abnormalities in 51.5% of patients with elevated values, along with histologic alterations in >70%.

Associations with rheumatologic disease activity were heterogeneous. In some cohorts, elevated FC paralleled higher BASDAI, BASFI, or ASDAS scores [9,13,20], whereas others [15,18] found no correlation. Kang et al [16] demonstrated that FC levels correlated with ASDAS–C-reactive protein (CRP), erythrocyte sedimentation rate (ESR) and enthesitis scores, suggesting utility beyond gut involvement.

Confounders and treatment effects were frequent. NSAID use was associated with higher FC [15,18], whereas patients on tumour necrosis factor (TNF) inhibitors tended to display lower values [10].

In their 2020 systematic review and meta-analysis, Ma et al [17] assessed the utility of FC as a biomarker in patients with SpA. The analysis included a broad range of studies, pooling data from ∼1000 patients. The authors found that FC was elevated in patients with SpA compared with healthy controls and correlated with disease activity. The association between FC and disease activity was variable, likely due to differences in study design, assay type, and confounding factors such as NSAID use. The review also highlighted the potential role of FC in differentiating inflammatory from noninflammatory back pain.

In a 2025 cross-sectional study, Arzuaga-Hernández et al [9] investigated the relationship between GI and psychological symptoms and FC levels in patients with SpA. Among 98 patients, elevated FC levels (>43.2 µg/g) were significantly associated with both higher GI symptom scores and markers of disease activity, including BASDAI and ASDAS. Endoscopic and histologic assessments confirmed a higher prevalence of gut inflammation in patients with elevated FC. Moreover, the study revealed a novel correlation between elevated FC and psychological parameters such as depression and feelings of helplessness, suggesting a possible link between intestinal inflammation and the gut–brain–joint axis in SpA.

The studies summarised in Table 1 highlight the consistent interest in FC as a noninvasive biomarker of gut inflammation in SpA. Most studies demonstrated elevated FC in patients with SpA compared with that in healthy controls, with moderate correlations to disease activity indices such as BASDAI and ASDAS. Several investigations confirmed associations between FC levels and magnetic resonance imaging or endoscopic evidence of inflammation. Screening strategies using FC thresholds (eg, 43.2-100 µg/g) improved early identification of subclinical IBD. Although NSAID exposure and microbiota composition influenced FC, treatment with TNF inhibitors tended to reduce its levels, supporting its potential role in disease monitoring and therapeutic guidance.

### Rheumatoid arthritis

Evidence in RA is more limited and is currently based on 2 studies (Table 3, Table 4) [21,22]. In a prospective early-RA cohort [21], patients had significantly elevated mean FC levels (∼71 µg/g) at diagnosis, which decreased after 3 months of treat-to-target therapy. Heidt et al [22] confirmed abnormal FC levels in 38% of patients with established RA, with higher prevalence in those receiving glucocorticoids. Although neither study included systematic endoscopic assessment, both suggest that subclinical gut inflammation may be present in a proportion of patients with RA and that FC may correlate with disease activity or treatment exposure.

**Table 3:** Study characteristics—RA

Reference, year	Country	Design	Aim of the study	Rheumatologic disease definition	Control	No. of patients	Age (y)	Male (%)	FC assay	Timing vs activity	GI symptoms	Endoscopy
Einarsson et al [21] (ACR), 2024	Sweden	Prospective cohort	Evaluate FC as biomarker in early RA and its response to therapy	Early RA (ACR/EULAR)	No	104	58	27	ELISA	Baseline + 3 mo	No	No
Heidt et al [22], 2023	Germany	Cross-sectional	Compare biomarkers of intestinal permeability (zonulin and LPS) and inflammation (FC and sCAL) in patients with RA	ACR/EULAR	No	61	63 (56-70.5)	33	ELISA kit (Immundiagnostik)	One-time sample	No	No

ACR, American College of Rheumatology; ELISA, enzyme-linked immunosorbent assay; EULAR, European Alliance of Associations for Rheumatology; FC, faecal calprotectin; GI, gastrointestinal; HC, healthy control; LPS, lipopolysacharides; RA, rheumatoid arthritis; sCAL, serum calprotectin.

**Table 4:** Summary of studies on FC in RA (2020-2025)

Reference, year	Normal FC values (µg/g)	FC values in RA (µg/g), median (range0	FC threshold (µg/g)	Association with digestive symptoms	Endoscopic/histologic correlation	Disease activity correlation	NSAIDs	Anti-TNFα	Key findings
Heidt et al [22], 2023	<50	36.1 (19.2-94)	NA	NA	NA	NA	NA	NA	Elevated FC/serum calprotectin in 38% of patients with RA; more frequent in those treated with glucocorticoids
Einarsson et al [21] (ACR), 2024	<50	71 (23-559) in early RA; 17 (1-138) in controls	>50	No	No	Correlated with DAS-28; decreased after T2T therapy	NA	NA	FC elevated at RA diagnosis; associated with disease activity and responsive to treatment

ACR, American College of Rheumatology; DAS-28, Disease Activity Score 28; FC, faecal calprotectin; NA, not applicable; NSAID, nonsteroidal anti-inflammatory drug; RA, rheumatoid arthritis; T2T, treat-to-target; TNF, tumour necrosis factor.

Evidence is currently insufficient to recommend routine FC screening in RA, but FC may help distinguish IBD from functional GI symptoms and could complement clinical assessment in early or refractory disease. In RA, emerging data indicate that FC may detect subclinical intestinal inflammation in early or active disease and could have a role in phenotyping patients, but robust prospective studies are needed.

Recently, Romero-Figueroa et al [23] discusses the emerging role of gut dysbiosis and intestinal inflammation in the pathogenesis of RA, with special attention to noninvasive biomarkers such as FC. This suggests a possible subclinical intestinal involvement in RA. The review underscores that FC is associated with increased intestinal permeability (potentially driven by zonulin) and may reflect local mucosal immune activation contributing to systemic inflammation.

The evidence for FC and serum calprotectin in RA is growing. Studies show elevated FC even in patients with early RA, correlating with clinical activity scores (DAS-28). FC may reflect increased intestinal permeability and dysbiosis, supporting a gut–joint axis hypothesis. Although these findings are promising, there is a need for standardisation in assays and thresholds to facilitate clinical integration.

### Fibromyalgia syndrome

Evidence in FMS is extremely limited and currently relies on a single cross-sectional study (Table 5, Table 6). Çınar et al. [24] compared patients with FMS with and without GI symptoms and reported significantly higher mean FC values in symptomatic patients (262.9 ± 181.2 µg/g) than in controls. The lack of correlation with histology and the absence of specificity to FMS limit conclusions. Findings likely reflect comorbid GI disorders in a subset of patients. Given that fibromyalgia is not classically considered an inflammatory disease, these findings warrant further investigation into potential gut–brain interactions and the role of low-grade inflammation in chronic pain syndromes.

**Table 5:** Study characteristics—FMS

Reference, year	Country	Design	Aim of the study	Rheumatologic disease definition	Control	No. of patients	Age (y), mean ± SD	Male (%)	FC assay	Timing vs activity	GI symptoms	Endoscopy
Çınar et al [24], 2023	Turkey	Cross-sectional	Assess intestinal inflammation in FMS with/without GI symptoms vs controls	ACR 2016 (65 FMS)	Yes (35 HC)	100	FMS: 46.9 ± 10.6; FMS + GI symptoms: 48.5 ± 11; controls: 39.2 ± 13.1 (lower, *P* < .05)	10	Calprest NG test (ELISA)	One-time sample	Yes	No

ACR, American College of Rheumatology; ELISA, enzyme-linked immunosorbent assay; FC, faecal calprotectin; FMS, fibromyalgia syndrome; GI, gastrointestinal; HC, healthy control

**Table 6:** Summary of studies on FC in FMS (2020-2025)

Reference, year	Normal FC values (µg/g)	FC values in FMS (µg/g)	FC threshold	Association with digestive symptoms	Endoscopic/histologic correlation	Disease activity correlation	NSAIDs	Anti-TNFα	Key findings
Çınar et al [24], 2023	<50	262.9 ± 181.2 µg/g	>50 µg/g	Yes – higher FC in FMS with GI symptoms vs FMS without GI symptoms and controls	No	NA	NA	NA	Elevated FC specifically in symptomatic FMS subgroup, suggesting gut involvement in a subset

FC, faecal calprotectin; FMS, fibromyalgia syndrome; GI, gastrointestinal; NA, not applicable; NSAID, nonsteroidal anti-inflammatory drug; TNF, tumour necrosis factor.

## DISCUSSION

The evidence synthesised in this review confirms that FC is a valuable noninvasive biomarker for identifying subclinical gut inflammation in patients with rheumatologic diseases.

### FC in SpA

FC measurement appears especially useful for identifying patients with occult gut inflammation, including those without overt GI symptoms. This may assist clinicians in selecting patients who warrant further endoscopic evaluation and could facilitate earlier recognition of IBD or related conditions.

Across studies, FC levels were significantly associated with established systemic inflammatory markers, including CRP, ESR, and ASDAS, supporting its role not only as a marker of mucosal inflammation but also as a potential indicator of global inflammatory burden. The observed reductions in FC levels under monoclonal anti–TNF-α therapy further suggest that effective systemic treatment may concomitantly improve intestinal inflammation. By contrast, the influence of NSAIDs on FC values remains inconsistent across studies. Together, these findings indicate that FC may provide complementary information in disease activity assessment and therapeutic monitoring.

Importantly, studies incorporating systematic endoscopic and histologic evaluation provide the strongest evidence for FC as a surrogate marker of subclinical IBD in SpA. In these cohorts, elevated FC frequently correlated with macroscopic or microscopic inflammation, even in the absence of digestive symptoms. However, such systematic assessments remain limited and are rarely performed outside SpA populations.

Emerging data also suggest associations between FC levels and extra-articular manifestations, including acute anterior uveitis and peripheral involvement, supporting the concept of a gut–joint axis and systemic immune activation [8]. Similarly, some studies [16] suggest a correlation between FC levels and peripheral joint or enthesis involvement, although this relationship remains less well defined. These observations support the concept of systemic immune activation, with the gut acting as a potential immunologic driver beyond axial inflammation.

Several studies have explored the relationship between gut microbiota composition and FC levels. Dysbiosis—characterised by altered microbial diversity and abundance—has been associated with increased FC and more active rheumatologic disease [6,25]. In axSpA, reduced levels of beneficial bacteria and increased proinflammatory species appear to parallel elevated FC levels, suggesting a microbiota–gut–joint axis. In some cases, microbial colonisation patterns have been linked to lower FC levels, highlighting the complexity of host–microbe interactions in inflammatory diseases [6,13].

### FC in RA

In RA, available data remain limited but suggest that FC may reflect persistent inflammatory activity, even in patients with normal conventional biomarkers. This observation is of particular interest in treat-to-target strategies, where detection of residual inflammation is clinically relevant. However, the absence of systematic endoscopic correlation in RA cohorts limits definitive conclusions regarding the intestinal origin of elevated FC.

### FC in FMS

Findings in FMS are preliminary. Although elevated FC has been reported in symptomatic subsets, the absence of consistent endoscopic confirmation and the high likelihood of functional GI overlap restrict interpretation. These observations raise hypotheses regarding gut–brain interactions and low-grade inflammation but currently lack sufficient evidence to support clinical implementation.

### Methodological limitations

Despite these promising findings, several important limitations must be acknowledged. Substantial heterogeneity exists in FC thresholds, with cutoffs ranging from ∼40 to 100 µg/g, limiting comparability and clinical applicability. Interindividual variability—related to age, body mass index, NSAID exposure, microbiome composition, and comorbidities—further reduces specificity when FC is used in isolation. Nonetheless, several studies suggest that lower FC thresholds may be associated with reduced specificity, whereas higher cutoffs improve predictive value for histologic inflammation or subsequent IBD diagnosis. These findings highlight the need for assay standardisation and clinically validated thresholds adapted to rheumatologic settings.

Moreover, the clinical context in which FC testing was performed varied widely, from systematic screening of unselected patients to symptom-driven assessment. In some cohorts, FC was measured systematically, whereas in others, it was performed only in patients with GI symptoms, suspected intestinal involvement, or uncontrolled inflammatory disease. This variability likely influenced the reported prevalence of elevated FC, further contributing to heterogeneity and limiting direct comparisons across cohorts.

Therapeutic exposure represents an additional and often underreported confounding factor. Interpretation of FC levels in rheumatologic populations should therefore always consider concomitant therapies, particularly NSAIDs, corticosteroids, biologic agents, and Janus kinase (JAK) inhibitors, as these treatments may substantially influence intestinal inflammation and FC concentrations independently of disease activity. Monoclonal anti–TNFα antibodies, which are effective in both rheumatologic diseases and IBD, are generally associated with reductions in FC levels and improvement of mucosal inflammation [9,17]. In contrast, the impact of other therapeutic classes may differ according to their mechanism of action. interleukin (IL)-17 inhibitors, although highly effective in axial and psoriatic disease, have been associated with incident or exacerbated IBD in susceptible patients, which may influence FC interpretation [[26], [27], [28], [29]]. Conversely, IL-23 inhibitors and JAK inhibitors have demonstrated efficacy in IBD and are associated with decreases in FC levels in several studies [30,31]. Data regarding methotrexate remain insufficient to draw firm conclusions [32]. Data regarding corticosteroids were scarce, but glucocorticoid exposure may also influence intestinal permeability and FC concentrations, representing another potential confounding factor [33]. Few studies have adjusted analyses for background therapies, which may partly explain variability in reported FC prevalence and diagnostic performance. Therefore, therapeutic exposure should be systematically considered when analysing FC results in rheumatologic populations, as it may represent a major confounding factor across studies.

The predominance of cross-sectional designs further limits interpretation. Longitudinal data evaluating FC dynamics in relation to disease progression, therapeutic response, or incident IBD remain scarce. Furthermore, the definition of intestinal inflammation or subclinical IBD lacks harmonisation across studies, ranging from clinical suspicion or biomarker elevation alone to endoscopic and histologic confirmation, thereby limiting comparability and external validity.

A formal pooled diagnostic accuracy analysis was not feasible given the marked heterogeneity in populations, methodologies, and outcome definitions. Consequently, conclusions regarding sensitivity and specificity remain descriptive.

### Clinical implications and future directions

Differentiating between functional GI symptoms (eg, irritable bowel syndrome–like features) and organic inflammation remains a clinical challenge [34,35]. FC has proven useful in this context: levels of <50 µg/g are generally considered reassuring in patients with FMS or RA presenting with GI complaints, while elevated values may prompt further investigation for IBD or subclinical gut involvement [34,36]. Thus, FC offers a noninvasive triage tool to avoid unnecessary colonoscopies in patients with functional symptoms and to prioritise endoscopy in those at higher risk of organic disease [37].

Comparative studies across rheumatologic conditions have demonstrated differences in baseline FC levels and their associations with disease activity [14,17,18]. In SpA and RA, elevated FC often reflects true mucosal inflammation, particularly when supported by imaging or endoscopy [8,14]. In contrast, in FMS, elevations are more modest and less consistently correlated with objective findings [24]. This suggests that FC may have higher specificity for inflammatory pathologies, although overlap exists. Understanding these disease-specific patterns is critical for interpreting FC results in clinical practice.

The integration of FC measurement into routine rheumatology practice could improve the early detection of IBD and subclinical gut inflammation, particularly in patients presenting with GI symptoms. However, accumulating evidence indicates that elevated FC levels may also be observed in patients without overt digestive complaints, reflecting subclinical intestinal inflammation that would otherwise remain undetected. In this context, FC may help identify patients at increased risk of intestinal involvement, facilitate risk stratification, and reduce unnecessary invasive procedures by guiding the selection of patients who may benefit most from endoscopic evaluation [13]. FC may also serve as a useful adjunct for monitoring therapeutic response, especially in patients receiving biologic therapies, as changes in FC levels may parallel both intestinal and systemic inflammatory activity. Nevertheless, widespread implementation of FC testing beyond symptom-driven indications requires standardisation of assay methods and consensus on optimal thresholds adapted to different rheumatologic populations to maximise clinical utility [33].

Future research should focus on large, prospective studies to validate the role of FC across diverse rheumatologic cohorts, in both symptomatic and asymptomatic patients. Such studies should investigate longitudinal relationships between FC levels, disease activity, and long-term outcomes, as well as the impact of different therapeutic strategies on gut inflammation. In fibromyalgia and other chronic pain syndromes, further exploration of the gut–brain axis and the contribution of low-grade intestinal inflammation may provide novel insights into disease mechanisms and potential therapeutic targets.

## CONCLUSIONS

FC emerges as a promising adjunct biomarker for identifying and monitoring intestinal involvement in rheumatologic diseases, particularly SpA. However, standardisation of assays and thresholds, systematic adjustment for therapeutic exposure, and well-designed prospective studies incorporating endoscopic validation are required before FC can be integrated into routine, guideline-driven clinical algorithms.

## CRediT authorship contribution statement

**Caroline Morizot:** Writing – review & editing, Writing – original draft, Validation, Methodology, Formal analysis, Data curation, Conceptualization. **Bénédicte Caron:** Writing – review & editing. **Jean-Yves Jouzeau:** Writing – review & editing. **David Moulin:** Writing – review & editing. **Patrick Netter:** Writing – review & editing. **Sailish Honap:** Writing – review & editing. **Damien Loeuille:** Writing – review & editing, Supervision, Methodology, Conceptualization. **Laurent Peyrin-Biroulet:** Writing – review & editing, Supervision, Methodology, Conceptualization.

## Competing interests

BC has received lecture and/or consulting fees from AbbVie, Amgen, Celltrion, Ferring, Galapagos, Janssen, Lilly, Nordic Pharma, Pfizer, and Takeda. SH has received speaker, consultant, and advisory board member fees and/or has received travel grants from Pfizer, Janssen, AbbVie, Takeda, Ferring, Lilly, Dr Falk Pharma, and Pharmacosmos. LPB has received consulting and/or lecture fees from AbbVie, Adacyte, Alimentiv, Alma Bio Therapeutics, Amgen, Applied Molecular Transport, Arena, Biogen, BMS, Celltrion, CONNECT Biopharm, Cytoki Pharma, Enthera, Ferring, Fresenius Kabi, Galapagos, Genentech, Gilead, Gossamer Bio, GSK, HAC-Pharma, IAG Image Analysis, Index Pharmaceuticals, Inotrem, Janssen, Lilly, Medac, Mopac, Morphic, MSD, Norgine, Nordic Pharma, Novartis, OM Pharma, ONO Pharma, OSE Immunotherapeutics, Pandion Therapeutics, Par’Immune, Pfizer, Prometheus, Protagonist, Roche, Sanofi, Sandoz, Takeda, Theravance, Thermo Fisher, Tigenix, Tillots, Viatris, Vifor, Ysopia, Abivax, Samsung, Ventyx, Roivant, and Vectivbio. The other authors report no conflicts of interest.
